# Three cases of African tick bite fever diagnosed in Quebec City: A case series

**DOI:** 10.1177/2050313X241260516

**Published:** 2024-06-14

**Authors:** Nicolas Caudrelier, Marie-Claude Dionne

**Affiliations:** Dermatology Division, Department of Medicine, CHU de Québec-Université Laval, Quebec City, QC, Canada

**Keywords:** African tick bite fever, rickettsiosis, spotted fever

## Abstract

African tick bite fever is a rickettsiosis of the spotted fever group that is endemic to sub-Saharan Africa and the Caribbean. It is characterized by eschars at the inoculation sites and a maculopapular rash which may be purpuric. We describe three cases that were diagnosed in Quebec City.

## Case series

### Case 1

**Figure 1.**
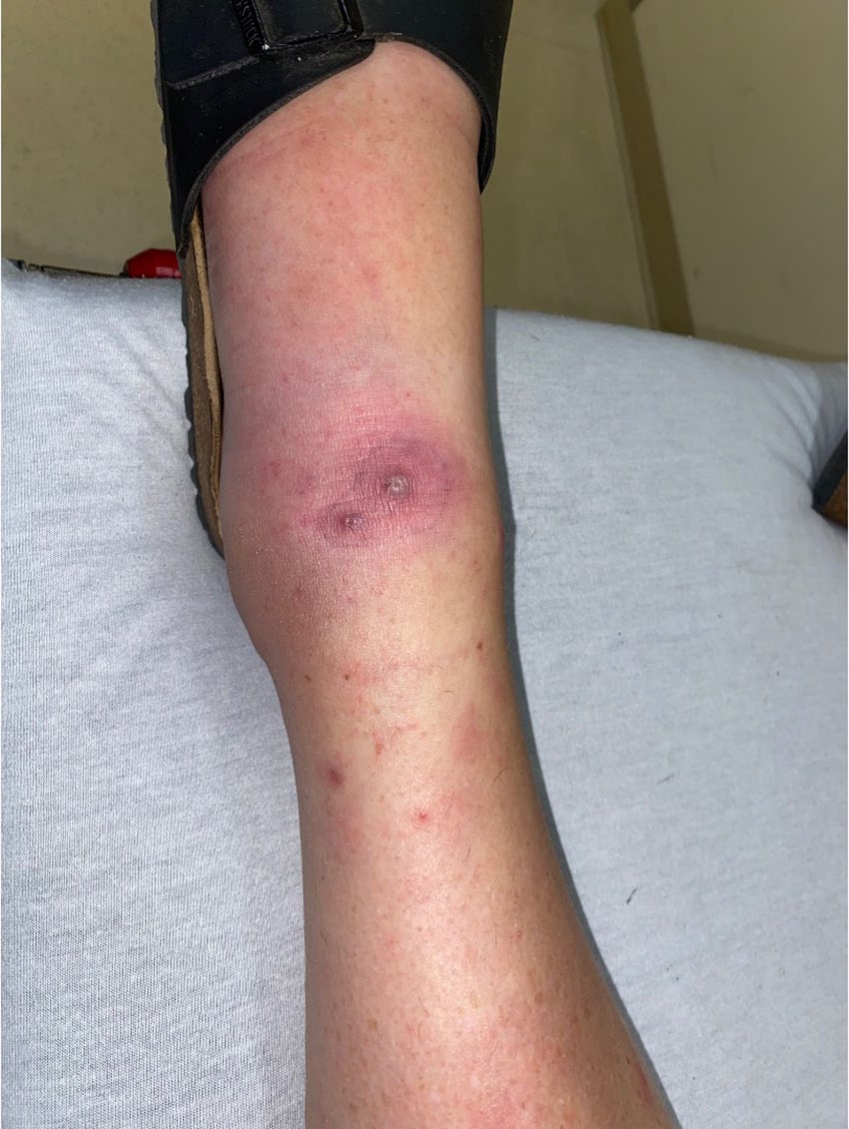
Case 1: erythematous eschar on the ankle with few purpuric macules and papules on the lower leg.

A 33-year-old woman, who returned from a trip to South Africa 3 days prior, presents to the emergency department with an eschar on her ankle (figure 1). She reports that this eschar has been present for 2 weeks. She also complains of a 1-week persistent fever. Ceftriaxone is started for suspicion of cellulitis. The patient returned 2 days later to the emergency for persistent fever and new maculopapular rash despite antibiotic treatment. The blood tests are unremarkable except for an increased C-reactive protein (48, normal <5). A dermatology consultation is requested. A rickettsia serology follows and proves to be in the gray zone (1/128). Two biopsies are done on the eschar. The polymerase chain reaction of the Rickettsia genus is positive, and histology demonstrates lymphomatoid vascular reaction. The skin lesions and fever resolve quickly with treatment with doxycycline.

### Case 2

**Figure 2.**
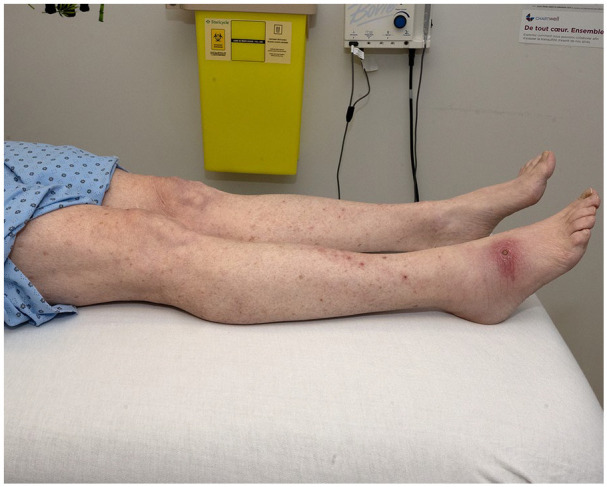
Case 2: eschar on the dorsal right foot with many erythematous edematous papules on the legs.

A 60-year-old woman consults at the emergency for a wound on her foot. She returned from a trip to South Africa 5 days prior. The ulcer has been present for 10 days, gradually increasing in size, and is associated with fever, headache, and myalgia. Physical examination demonstrates a centimeter-sized necrotic ulcer surrounded by erythema, edema, and warmth on her foot. The patient also presents with a few erythematous papules and petechiae on the limbs and back (figure2). Routine blood tests are unremarkable. A dermatology consultation is requested. Biopsies are taken on papules on the leg, demonstrating lymphocytic vasculitis. Rickettsia PCR on skin tissue is negative, but Rickettsia serology is positive (1/64). Symptoms and rash improve within a few days of doxycycline and cefadroxil.

### Case 3

**Figure 3.**
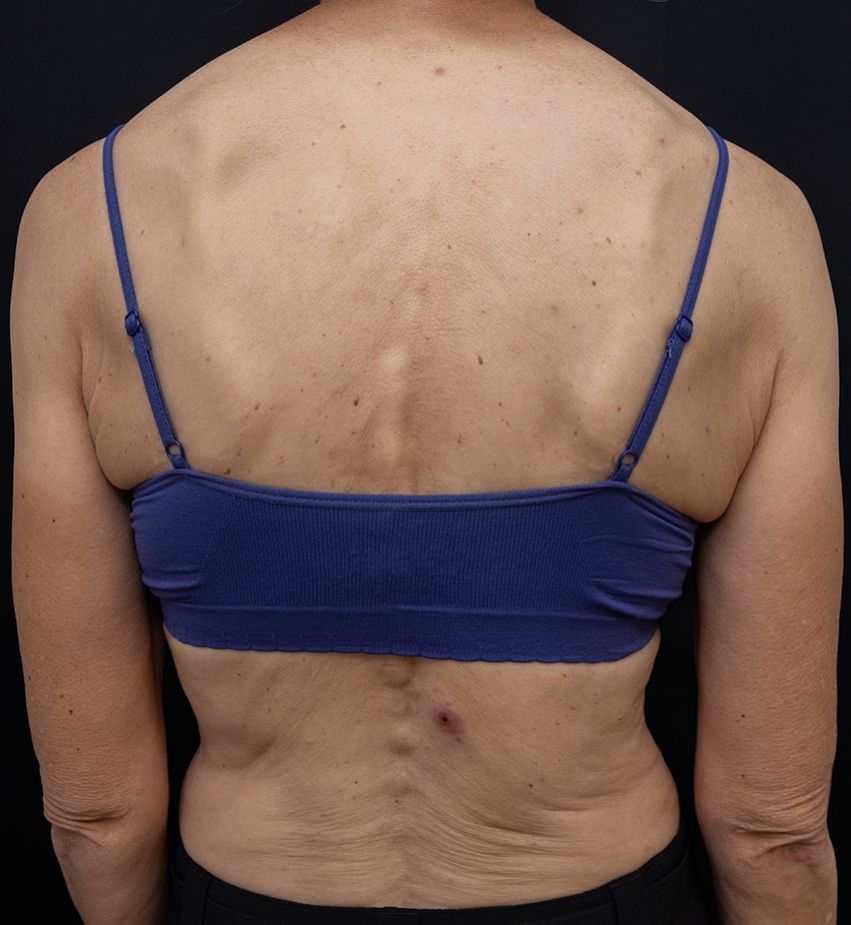
Case 3: eschar on the lower back.

**Figure 4.**
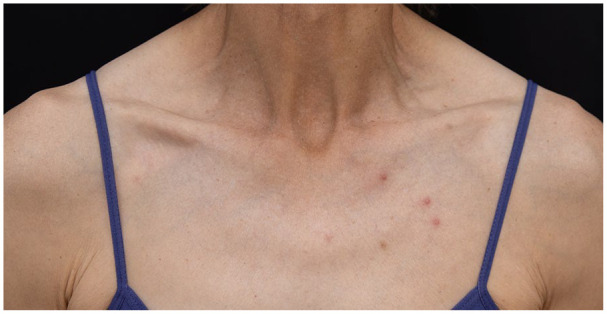
Case 3: few erythematous papules on the trunk.

A 61-year-old woman presents to the emergency department with a rash and a tender lump in the armpit. She returned 13 days earlier from South Africa. She has a sensitive papule on her back for 1 week that has become necrotic and has a sensitive axillary lymphadenopathy. She also presented a rash of asymptomatic papules on the upper limbs and trunk, and a light fever for the past 2 days (figures 3 and 4). Her partner with whom she traveled was bitten by a tick and was diagnosed with Rickettsiosis. Routine blood tests are normal. In the dermatology department, skin biopsies are performed on the eschar, demonstrating an intense lymphomatoid vascular reaction associated with focal dermal necrosis. Rickettsia PCR on biopsy is positive, while PCR in blood is negative. The eruption and fever vanish rapidly with doxycycline.

## Discussion

Rickettsioses are a group of diseases caused by Gram-negative intracellular bacteria that live within an arthropod.^1^ African tick bite fever is part of the spotted fever group, which are transmitted by arthropods during their feeding.^1^ African tick bite fever (ATBF) is caused by the bacteria *Rickettsia africae*.^1^ It is transmitted by two ticks: *Amblyomma variegatum* and *Amblyomma hebraeum*, which are aggressive ticks that are endemic to sub-Saharan Africa and the Caribbean.^1^ It is the most prevalent rickettsiosis in humans and the second most common cause of return-from-travel fever in sub-Saharan Africa after malaria.^2^ It manifests first by inoculation eschar which can be multiple, then by flu-like syndrome, and a generalized rash a few days later.^1^ It is a mild rickettsiosis, and no fatal cases have been described.^1^ It is diagnosed based on clinical presentation and compatible epidemiology.^3^ PCR on eschar biopsy is the most sensitive test to confirm the diagnosis.^3^ Like other rickettsioses, ATBF is treated with doxycycline and is to be started empirically in case of diagnostic suspicion.^3^

We presented three cases of classic ATBF diagnosed in Quebec City. Interestingly, none of our cases presented multiple eschars although this is reported in 50% of cases in the literature.^2^ It is pathognomonic of ATBF, because the ticks which transmit the bacteria are very aggressive, and patients may be bitten by multiple ticks simultaneously.^2^ Furthermore, our patients presented with mild illnesses, which is consistent with the literature.^4^ Surprisingly, none of our patients presented with hepatic transaminitis or blood count abnormalities (leukopenia, thrombocytopenia) even though these are common findings.^2^ In addition, all our patients had their diagnosis confirmed by PCR on skin biopsy or serology. One of our patients had a negative biopsy PCR even though it is the most sensitive diagnostic method. However, the biopsy was performed on a papule other than the eschar, which may explain lower sensitivity and a negative result. However, for this patient, the positive serology confirmed the diagnosis of ATBF. Physicians should consider using multiple diagnostic methods to increase their diagnostic acuity. Ultimately, all our patients healed quickly on doxycycline, which is the treatment of choice for rickettsioses.^3^

In conclusion, we present three cases of ATBF in travelers returning to Quebec City. Dermatologists and primary care doctors should remain cautious as this is the most common rickettsial disease worldwide, and we can expect to see more as international tourism increases.
